# International Health Regulations—What Gets Measured Gets Done

**DOI:** 10.3201/eid1807.120487

**Published:** 2012-07

**Authors:** Kashef Ijaz, Eric Kasowski, Ray R. Arthur, Frederick J. Angulo, Scott F. Dowell

**Affiliations:** World Health Organization Collaborating Center for International Health Regulations Implementation of National Surveillance and Response Capacity, Atlanta, Georgia, USA; and; Centers for Disease Control and Prevention, Atlanta

**Keywords:** International Health Regulations, monitoring and evaluation, implementation, disease notification, public health, epidemiology

## Abstract

The global spread of severe acute respiratory syndrome highlighted the need to detect and control disease outbreaks at their source, as envisioned by the 2005 revised International Health Regulations (IHR). June 2012 marked the initial deadline by which all 194 World Health Organization (WHO) member states agreed to have IHR core capacities fully implemented for limiting the spread of public health emergencies of international concern. Many countries fell short of these implementation goals and requested a 2-year extension. The degree to which achieving IHR compliance will result in global health security is not clear, but what is clear is that progress against the threat of epidemic disease requires a focused approach that can be monitored and measured efficiently. We developed concrete goals and metrics for 4 of the 8 core capacities with other US government partners in consultation with WHO and national collaborators worldwide. The intent is to offer an example of an approach to implementing and monitoring IHR for consideration or adaptation by countries that complements other frameworks and goals of IHR. Without concrete metrics, IHR may waste its considerable promise as an instrument for global health security against public health emergencies.

## Abstract 2

Focus on goals and metrics for 4 core capacities illustrates 1 approach to implementing IHR.

The global spread of severe acute respiratory syndrome highlighted the need to detect and control disease outbreaks at their source (*1**,**2*). The 2005 revised International Health Regulations (IHR) were established as a legally binding agreement providing a framework for improving detection, reporting, and response to public health emergencies of international concern (public health emergencies) (*3*). The global implementation of IHR began on June 15, 2007, and in an unusual episode of international consensus, all 194 WHO member states ratified the agreement. When implemented, IHR should improve global capacity to detect, assess, notify, and respond to public health threats. Properly and fully implemented, IHR should usher in a new global era of international communication, cooperation, and unprecedented security against the epidemic threats that have plagued humanity since ancient times. But there is a problem.

After enactment of the revised IHR in June 2007, all member countries were required to develop and implement a minimum of core public health capacities by June 2012, the 5-year anniversary of IHR’s enforcement. Many countries did not meet the deadline and have requested a 2-year extension. In an era of limited resources, competing priorities, and political challenges, achievement of the IHR implementation goals, even with an extension, will be a challenge. Focusing efforts toward IHR implementation and capacity building and enabling all countries to measure progress toward IHR implementation is, therefore, essential. Toward this end, concrete goals and metrics for 4 of the 8 core capacities were developed by the WHO Collaborating Center for IHR Implementation of National Surveillance and Response Capacity at the Centers for Disease Control and Prevention with other US government partners in consultation with WHO and national collaborators worldwide (Table 1). This approach is in alignment with WHO’s IHR framework and facilitates measurement of implementation activities. The framework focuses on 4 of the core capacities (human resources, surveillance, laboratory, and response) and builds on WHO’s IHR Monitoring Framework by defining simple standards for these capacities (*4*). The focus on these 4 capacities should not imply that they are more important than other capacities (legislation, policy, and financing; coordination; advocacy and national focal point communications; preparedness; and risk communication) because implementation of IHR requires implementation of all 8 capacities. The intent is to assist partner countries in better focusing efforts, to improving efficiency at IHR implementation, and to better monitoring and evaluating progress. Focusing on the subset of IHR core capacities also will provide a foundation for an all-hazards approach for addressing public health emergencies regardless of cause. We describe the rationale, targets, and definitions for these 4 goals and means by which countries can use the data collected through monitoring and evaluation indicators for measuring progress related to these 4 core capacities.

**Table 1:** Goals, targets, and intended use for 4 core capacities for focusing International Health Regulations implementation

Capacity	Goal	Target/measure	Intended use
Human resources	Ensure adequate numbers of trained personnel are available to support the response to a public health emergency	A national workforce plan and 1 trained field epidemiologist for every 200,000 persons	Document that a workforce plan exists and is maintained and updated, and monitor annual progress toward the goal of 1 trained field epidemiologist for every 200,000 persons.
Surveillance	Ensure that surveillance systems capable of detecting selected potential public health emergencies in any part of the country are established and functioning	Surveillance infrastructure that demonstrates the ability to detect >3 of 5 syndromes indicative of a potential public health emergency of international concern	Monitor and evaluate the effectiveness of the surveillance system, and identify areas for improvement within the country’s public health surveillance infrastructure.
Laboratory	Ensure access to laboratory diagnostic capabilities that can identify a range of emerging epidemic pathogens by using the full spectrum of basic laboratory testing methods	Ability to perform 10 core diagnostic tests for confirmation of indicator pathogens from any part of the country	Assess/measure capacity for detection will by using external/internal quality assurance for each of the 10 core tests and indicator pathogens using standard methods.
Response	Ensure countries have adequate rapid response capacity for public health emergencies	At least 1 functioning rapid response team per major administrative unit	Maintain an adequate number of rapid response teams with the necessary training, appropriate personnel, and regular outbreak responses.

## Human Resources

A well-trained cadre of public health professionals at the national health authorities at a country’s central and local levels is needed for timely detection and response to public health emergencies. There is a worldwide shortage of public health professionals who are trained in public health practice and have had competency-based public health field experience. Building the cadre of field-trained epidemiologists available to monitor disease trends, inform decision makers about potential disease threats, and guide response during a public health emergency should be one of the first priorities in implementing the IHR.

The aim of the human resource goal is to ensure adequate numbers of trained personnel for response to a public health emergency. Specific targets to measure progress toward completion of this goal are a fully adopted national workforce plan and >1 trained field epidemiologist per 200,000 population who are active in the public health sector (*5*). Although the workforce plan cannot ensure that trained professionals remain in the public health sector, it will at least indicate a government’s commitment to public health through stability of the public health workforce. These concrete indicators enable measurement of incremental progress and are specific enough to enable tracking of success and clear documentation of failure.

## Surveillance

Disease surveillance is a cornerstone of public health practice. It provides for systematic and ongoing collection of data that help identify and detect disease-related aberrations that might constitute public health emergencies. Additionally, surveillance for key disease syndromes provides the foundation for interpreting signals of possible emergencies and early notification of outbreaks of potentially devastating diseases (*6*). The following 5 syndromes have internationally recognized standards for syndromic surveillance: severe acute respiratory syndrome, acute neurologic syndrome, acute hemorrhagic fever, acute watery diarrhea with dehydration, and jaundice with fever (*7**,**8*).

The metrics focus on the ability to detect public health emergencies with a target of documenting that >3 of these syndromes have surveillance systems in place that meet the respective international standards. These metrics will assist countries in ensuring that efforts at disease surveillance are effective and that systemic incentives are appropriately aligned to provide early warning for a potential public health emergency. The 3 syndromes chosen will depend on national disease control priorities. These surveillance systems should include early warning surveillance data and laboratory findings, which should be analyzed by trained epidemiologists.

Information for syndromic surveillance collected at the clinic or hospital level can help generate village- and district-level alerts. An alert investigation unit can then investigate these alerts, including an in-depth epidemiologic analysis. On the basis of the outcome of the analysis, rapid response teams can be deployed to respond to a public health event or outbreak.

## Laboratory

Laboratory diagnostic capacity can help in detecting emerging or reemerging pathogens in a timely manner and can support syndromic surveillance systems by adding specificity. Given the costs associated with establishing laboratory diagnostic capacity, diagnostic capability might not be feasible for all pathogens for every country. Therefore, pooling international laboratory resources through networks of local, national, regional, and international reference laboratories is encouraged. However, countries should be able to provide certain core diagnostic tests (either through their own or through network capacity) quickly and reliably to direct disease surveillance and response activities.

The metrics focus on the ability to perform 10 international reference standard tests for patients from any part of the country. The core tests and their respective indicator pathogens are selected from the IHR immediately notifiable list, the WHO Top Ten Causes of Death in low-income countries (www.who.int/mediacentre/factsheets/fs310/en/index.html), and tests and indicator pathogens selected by the country on the basis of major national public health concern (Table 2).

**Table 2:** Core laboratory tests and indicator pathogens in the International Health Regulations

Core test	Indicator pathogen	Turnaround time from receipt in the laboratory
PCR	Influenza virus*	Within 24 h
Virus culture	Poliovirus*	Within 14 d
Serology	HIV†	Within 5 d
Microscopy	*Mycobacterium tuberculosis†*	Within 3 d
Rapid diagnostic test	*Plasmodium* spp.†	Within 2 h
Bacterial culture	*Salmonella enteritidis* serotype Typhi‡	Within 3 d
Local priority test	Local priority test§	Local priority test
Local priority test	Local priority test§	Local priority test
Local priority test	Local priority test§	Local priority test
Local priority test	Local priority test§	Local priority test

*Selected from the International Health Regulations immediately notifiable list. †Selected from WHO Top Ten Causes of Death in low-income countries (www.who.int/mediacentre/factsheets/fs310/en/index.html). ‡Selected from WHO Global Foodborne Infections Network (*9*). §Indicator pathogens selected by the country on the basis of major national public health concern.

However, achievement of laboratory diagnostic capacity requires all major components of the laboratory network to be well integrated in the national laboratory system. Components of such a system include sample collection, specimen transport, specimen processing, quality management systems, biosafety and biosecurity (specimen storage), staff, infrastructure, cold chains, reporting, and networking peripheral and central or regional reference laboratories. Data on the capacity and ability of the country to perform and report the 10 core tests can be used to monitor the ability of a country’s own laboratories or the reference laboratories to which it sends specimens to confirm and characterize these indicator pathogens and identify areas for improvement.

## Response

To implement IHR 2005, countries must have adequate rapid response capacity. During a public health emergency, timely response to public health events and threats is essential to prevent excess illness and death and control further transmission, including transborder spread. The presence of well-trained and functioning rapid response teams at local and national levels in a country can ensure a rapid, well-coordinated, and organized public health response.

These rapid response units should comprise a multidisciplinary team of trained public health professionals—medical epidemiologists, veterinarians, laboratory scientists, clinicians, chemical experts, and radiologic experts—as appropriate for the event who routinely deploy within 24 hours after a reported event. Rapid response units enhance a country’s ability to respond to outbreaks in a timely and effective manner.

These teams should undergo regular exercises for responding to public health emergency events, including >2 field outbreak investigations per year. They also should be trained in the 10 basic steps for outbreak investigations (*10*).

To meet the goal of adequate response capacity for public health emergencies, we propose a target of >1 functioning rapid response team per major administrative unit (district, province, or state). Larger administrative areas might need >1 team. Data and after-action reports from outbreak responses collected annually will enable the countries to monitor their progress, identify gaps, and improve performance.

## Conclusions

Implementation of IHR, required of all WHO member states, was not completed by the June 2012 deadline. The aim is for all countries to develop or enhance the ability to detect and respond to public health emergencies. Additionally, possible public health emergencies of international concern also need to be reported to prevent the spread of disease around the globe. Countries need concrete and well-defined goals and indicators to monitor their progress toward implementation of IHR core capacities. Even though we described metrics for 4 of the 8 IHR core capacities, we emphasize that full IHR compliance requires implementation of all 8 capacities. Goals and progress indicators also might be useful for the other 4 capacities. Without explicit goals and targets, the promise of international consensus around IHR might be wasted, but with them there is hope that what gets measured will eventually get done.
